# Self-cut titanium-coated polypropylene mesh versus pre-cut mesh-kit for transvaginal treatment of severe pelvic organ prolapse: study protocol for a multicenter non-inferiority trial

**DOI:** 10.1186/s13063-019-3966-3

**Published:** 2020-02-26

**Authors:** Juan Chen, Jiajie Yu, Abraham Morse, Christian Fünfgeld, Kuanhui Huang, Jian Gong, Guangshi Tao, Binan Wang, Yuling Wang, Xiangyang Jiang, Gulina Ababaikeli, Peishu Liu, Hatiguli Nisier, Xiaowei Zhang, Ping Wang, Xin Sun, Lan Zhu

**Affiliations:** 1 Department of Obstetrics/Gynecology, Peking Union Medical College Hospital, Chinese Academy of Medical Science, No. 1 Shuaifuyuan, Dongcheng District, Beijing, 100730 China; 2 Chinese Evidence-based Medicine Centre, West China Hospital, Sichuan University, Chengdu, Sichuan China; 3 Guangzhou Women and Children’s Medical Center, Guangzhou, China; 4 Center for surgery of Pelvic Prolapse and Incontinence, Tettnang Hospital (Klinik Tettnang GmbH), Tettnang, Germany; 5 Department of Obstetrics and Gynecology, Xiamen Chang Gung Memorial Hospital, Xiamen, Fujian China; 6 Department of Obstetrics/Gynecology, the Affiliated Wuxi Maternity and Child Health Care Hospital of Nanjing Medical University Hospital, Wuxi, Jiangsu China; 7 Department of Obstetrics/Gynecology, The Second Xiangya Hospital of Central South University Changsha, Hunan, China; 8 Department of Obstetrics/Gynecology, Changsha Hospital for Maternal and Child Health Care, Changsha, Hunan China; 9 Department of Obstetrics/Gynecology, Foshan Maternal and Child Health Care Hospital, Foshan, Guangdong China; 10 Department of Gynecology, Shaanxi Provincial People’s Hospital, Xi’an, Shanxi China; 11 Department of Obstetrics/Gynecology, The First Affiliated Hospital of Xinjiang Medical University Ürümqi, Xinjiang, China; 12 Department of Obstetrics/Gynecology, Qilu Hospital of Shandong University, Jinan, Shandong China; 13 Department of Obstetrics/Gynecology, The People’s Hospital of Xinjiang Uygur Autonomous Region Ürümqi, Xinjiang, China; 14 Department of Obstetrics/Gynecology, the First Affiliated Hospital of Guangzhou Medical University, Guangzhou, Guangdong China; 15 Department of Obstetrics/Gynecology, Sichuan University West China Second University Hospital, Chengdu, China; 16 Chinese Evidence-based Medicine Center and National Clinical Research Center for Geriatrics, West China Hospital, Sichuan University and Collaborative Innovation Center, Chengdu, Sichuan China

**Keywords:** Pelvic organ prolapse, Transvaginal mesh, Non-inferiority

## Abstract

**Background:**

Pelvic organ prolapse (POP) is a common health problem and has significant negative effects on a woman’s quality of life. The transvaginal mesh procedure is a durable reconstructive surgery, but the mesh kits are expensive for underdeveloped countries. Our previous case-series study showed that the use of self-cut mesh had a good success rate (91.8% at 1-year follow-up) and low complication rate. This trial is designed to compare a self-cut titanium-coated polypropylene mesh procedure with a mesh kit for the treatment of symptomatic stage III–IV anterior or apical prolapse in terms of efficacy, safety and cost-effectiveness.

**Methods:**

The trial is a randomized controlled multicenter non-inferiority trial. The primary outcome measure is the composite success rate at 1-year follow-up. The secondary outcomes are anatomic outcomes of each vaginal segment (anterior, posterior and apical) using the POP-Q score, subjective improvement of quality of life according to questionnaires, intraoperative parameters, complications and costs. Analysis will be performed according to the intention-to-treat principle. Based on a comparable success rate of 90% and 10% as the margin (β = 0.2 and one-sided α = 0.025), about 312 patients in total from 11 centers will be recruited including 10% dropout. The aims of the research are to demonstrate whether the self-cut mesh procedure is non-inferior to the mesh-kit procedure and to investigate the performance of titanium-coated mesh for vaginal prolapse repair.

**Discussion:**

This multicenter non-inferiority trial will evaluate whether the efficacy and safety of self-cut mesh is non-inferior to mesh kits in women with severe symptomatic stage III–IV anterior or apical prolapse. If we are able to show that the self-cut mesh procedure is non-inferior to the mesh-kit procedure in success rates, then the self-cut mesh procedure may be more cost-effective.

**Trial registration:**

ClinicalTrials.gov, NCT03283124. Registered on 17 January 2018.

## Background

Pelvic organ prolapse (POP) is a common health problem and has significant negative effects on a woman’s quality of life. The prevalence of symptomatic POP in China is about 9.6% according to our national epidemiology study (unpublished). Lower wealth status maybe one of the risk factors for POP [1, 2]. Professional society guidelines indicated that transvaginal mesh (TVM) repair should be reserved for high-risk patients, such as individuals with recurrent prolapse (particularly of the anterior segment) or with medical comorbidity that precludes more invasive and lengthier abdominal procedures [3]. The consensus statement in China also proposed that transvaginal polypropylene mesh repair (either commercial pre-cut mesh devices or self-cut mesh) was most appropriate for severe POP (stage III–IV) and recurrent POP [4]. However, the high cost associated with available commercial mesh kits in China (approximately 25,000 RMB) poses a significant challenge for non-directive surgical counseling. Because POP patients in our practice typically have a combination of anterior and apical prolapse, we designed a TVM system in 2006 which included specially designed reusable trocars and self-cut mesh [5]. The mesh pieces used in surgery were cut from a single piece of polypropylene mesh (10 cm × 15 cm GyneMesh; Ethicon, Somerville, NJ, USA). A 7-year prospective cohort study indicated that self-cut TVM repair had a good long-term results, with 84.3% anatomic success (POP-Q stage 0 or I) and 8.9% mesh-related complications [6]. This result was in line with mesh-kit surgical repair for POP reported by other surgeons. From 2006 to 2008, the gynecology department of Peking Union Hospital in Beijing, China conducted a multicenter prospective trial to evaluate the anatomic and quality-of-life outcomes for treatment of severe POP with self-cut TVM repair [7]. In this prospective case series, the anatomical success was 91.7%, and there were clinically and statistically significant improvements in quality of life. The mesh exposure or erosion rate was 6.9%. It appeared that our TVM procedure with self-cut mesh was safe and effective in treatment of severe POP with less cost when compared with mesh-kit procedures.

Titanium-coated meshes are new products in POP repair, which improved the pelvic floor-related quality of life and sexual function in a prospective multicenter trial [8]. Fünfgeld et al. [9] reported a large prospective multicenter study in Germany, with 289 patients who underwent surgery with a titanium-coated polypropylene mesh-kit (TiLOOP® Total 6; pfm medical ag, Germany) and were followed up for a median of 36 months. The recurrence rate for the anterior compartment was 4.5%, and the quality of life improved significantly. The erosion rate was 10.5% (30/286). From August 2015, the Department of Gynecology at Peking Union Medical College Hospital, Beijing, China began to use titanium-coated mesh and we reported the results of 18 patients, who followed up at a mean of 10.9 months (4–17 months) with an objective success rate of 100% and no exposure of the mesh or erosion after surgery [10].

We did not find studies comparing self-cut titanium-coated mesh procedures with mesh-kit procedures in patients with POP. This study aims to demonstrate whether the self-cut mesh procedure is non-inferior to the mesh-kit procedure and to investigate the performance of titanium-coated mesh for vaginal prolapse repair.

## Methods/design

### Study objectives

This RCT aims to compare the outcomes of self-cut versus mesh-kit titanium-coated polypropylene transvaginal mesh repair in the treatment of POP. The primary outcome is the composite success rate at 1 year, and the secondary outcomes include perioperative parameters, disease-specific quality of life, sexual function, complications and costs. We have developed the following hypotheses:

- The composite outcome of TVM repair using self-cut mesh is non-inferior to TVM repair using a mesh kit.
- TVM repair using self-cut mesh is non-inferior to the TVM repair using a mesh kit in disease-specific quality of life, sexual function scores and complications.
- The TVM repair using self-cut mesh has lower total hospital costs than TVM repair using a mesh kit.

### Overview of study design

The trial is a multicenter randomized controlled non-inferiority trial. The study protocol and informed consent was approved by the Institutional Review Board at Peking Union Medical College Hospital. The trial was registered with www.clinicaltrials.gov (NCT03283124).

The trial will recruit patients from 11 tertiary hospitals in China. The gynecology department at each participating hospital should perform at least 50 POP surgeries each year. An electronic data capture (EDC) system is developed with a contract research organization (CRO), which is responsible for data management. The CRO will have no role in the analysis of the data or the eventual production of any research manuscripts.

**Fig. 1:**
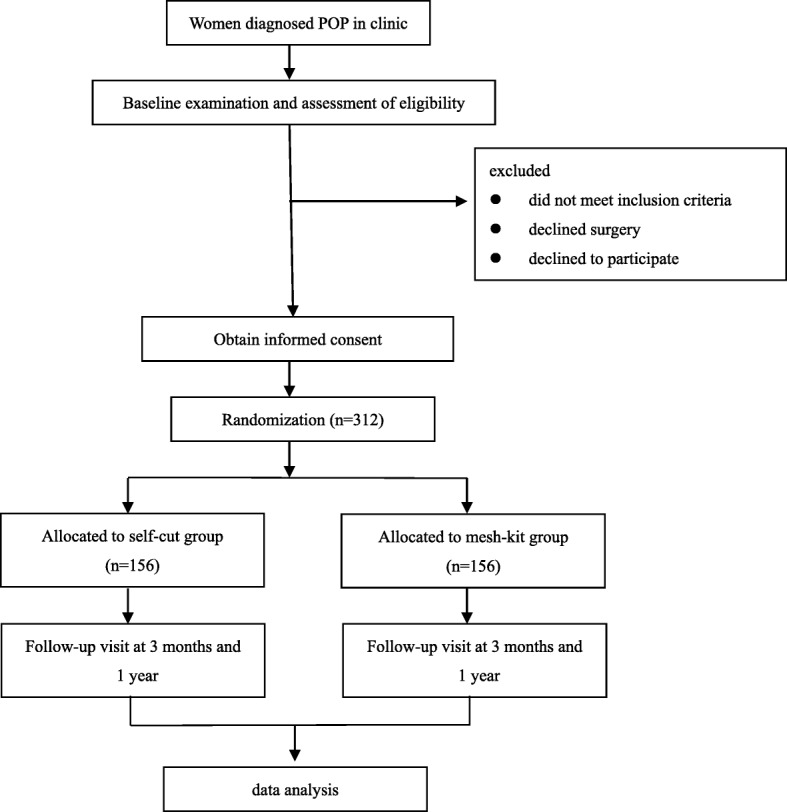
Study design. POP pelvic organ prolapse

**Fig. 2:**
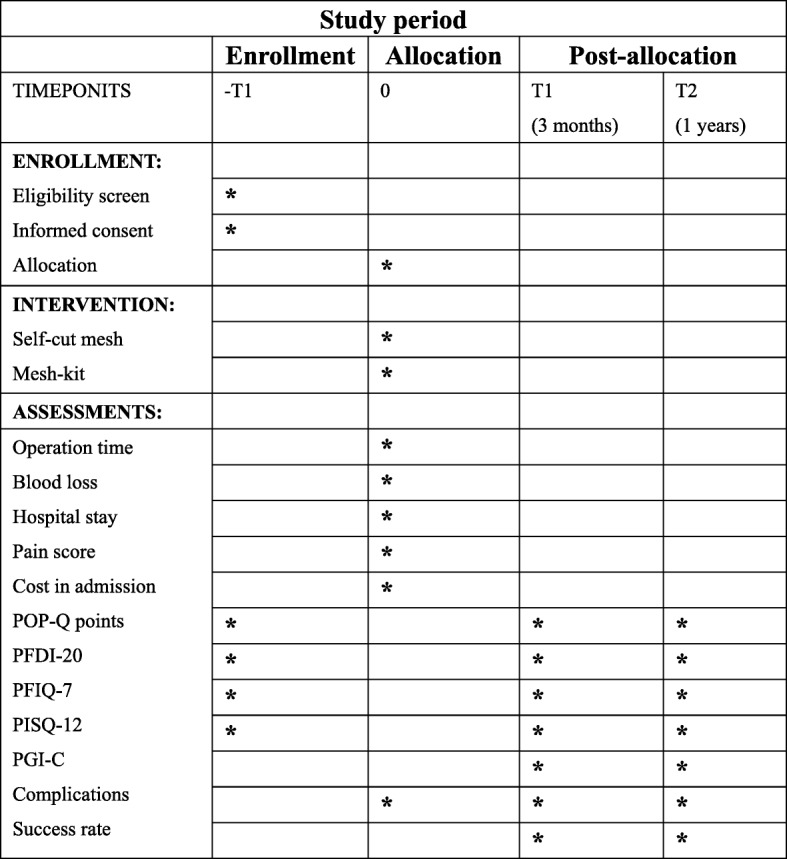
Study flow chart. PFDI-20 Pelvic Floor Distress Inventory-20, PFIQ-7 Pelvic Floor Impact Questionnaire short form, PGI-C Patient Global Impression of Change, PISQ-12 Pelvic Organ Prolapse/Urinary Incontinence Sexual Questionnaire short form, POP-Q Pelvic Organ Prolapse Quantification (system)

All eligible women at each center will be invited to participate. It is not possible to blind surgeons to the allocated surgical procedure. Women are not able to be blinded because the cost of the implant is paid out of pocket, and they will be informed about the difference in cost between the two mesh products as part of the consent process. An independent staff member or a research nurse who is not involved in treatment is blinded and will carry out the questionnaire collection and follow-up POP-Q measurement. The planned visit and examination schedule is presented in Figs. 1 and 2 (see Additional file 1).

### Participating hospitals

The trial will be performed at 11 hospitals in China: Peking Union Medical College Hospital; Wuxi Maternal and Child Health Care Hospital; Changsha Maternal and Child Health Care Hospital; Foshan Maternal and Child Health Care Hospital; The First Affiliated Hospital of Guangzhou Medical College; The Second Xiangya Hospital of Central South University; Qilu Hospital of Shandong University; Shanxi Provincial People’s Hospital; Sichuan University West China Second University Hospital; The First Affiliated Hospital of Xinjiang Medical University; and The People’s Hospital of Xinjiang Uygur Autonomous Region.

In order to minimize performance bias, only surgeons with adequate experience in TVM procedures (more than 20 cases per year) will serve as the primary surgeon in this trial. Since the only difference between the two arms involves modest differences in the equipment used, all surgeons will be able to perform both procedures. POP diagnosis, POP-Q measurement, questionnaires and standardized procedures will be trained in a standardized fashion. To ensure standardization for all participating centers, a video containing the crucial steps of the consent process will be used.

### Study population and recruitment

We will include the patient if she meets all of the following criteria:

- Symptomatic POP with apical and/or anterior vaginal prolapse stage III–IV—only patients with moderate posterior vaginal prolapsed stage I–II (C > + 1 cm or Ba > + 1 cm, with Bp ≤ + 1 cm by the POP-Q) will be included; those with stage III–IV posterior prolapse will be excluded; both primary and recurrent POP patients may be enrolled
- Must be more than 3 years after menopause or aged more than 55 years and less than 75 years
- Chooses transvaginal mesh treatment after appropriate surgical counseling
- Is willing and able to comply with the follow-up regimen
- Is capable of providing informed consent

We will exclude the patient if she meets any of the following criteria:

- High surgical risk due to medical comorbidities, such as active gynecologic and urinary tract infection, anticoagulation treatment or coagulation disorders, prior pelvic radiation therapy, neurologic or medical condition affecting bladder and bowel function (e.g., multiple sclerosis, spinal cord injury or stroke with residual neurologic deficit), chronic pelvic pain
- The need for concomitant anti-incontinence procedure, as we would like to minimize the influence of other procedures

Women eligible for this trial will be informed about the study objectives, designs, methods, potential advantages and limitations of the treatment. They can refuse or withdraw at any time with no consequences for their treatment. Before randomization, written informed consent will be obtained from each patient.

### Primary and secondary outcomes

The primary outcome measure is a composite surgical success variable measured at 1 year after surgery, defined as follows:

1. Absence of vaginal bulge symptoms as indicated by a rating of 0 on question 3 of the Pelvic Floor Distress Inventory-20 (PFDI-20): “Do you usually have a bulge or something falling out that you can see or feel in your vaginal area?”
2. No additional re-treatment (surgical or not) for POP
3. No POP-Q point at or beyond the hymen (i.e., Aa, Ba, C, Ap, Bp all < 0 cm)

The secondary outcomes include the following:

1. Anatomic outcome (POP-Q score) of each vaginal segment
2. Symptomatic improvement — relief of symptoms of pelvic floor disorders, including urinary, bowel and sexual function using validated instruments
3. Intraoperative parameters
4. Complications
5. Costs, defined as the direct total charges for the surgical admission including operation, medication and use of materials (e.g., surgical mesh)

### Randomization

After informed consent is signed, the patient will be registered on the web-based EDC system by the research staff to allocate each a unique study number prior to randomization. Research staff will access the system and request randomization, using the study number and initials. Patients are randomized in a 1:1 ratio to either the “self-cut mesh” group or the “mesh-kit” group according to a computer-generated randomization sequence with a block size of six. Randomization will be stratified according to centers. The patient and surgeon will be informed about the allocated operative procedure after the randomization.

### Data collection

Age, parity, body mass index, smoking history, time since menopause, use of hormone replacement therapy, medical and obstetric history, previous pelvic floor and gynecological surgery will be recorded. All patients will undergo routine pelvic examination, which includes routine bimanual examination, and vaginal inspection in a 45° semi-upright position for staging uterovaginal prolapse by POP-Q on maximum Valsalva effort in the lithotomy position. Routine ultrasound examination to exclude uterine or ovarian disease and cervical screening will be performed to exclude high-risk cervical dysplasia. A 1-h pad test and occult stress urinary incontinence test and uroflowmetry will be administered to all participants.

Patients will complete four questionnaires. The Chinese version of the Pelvic Floor Impact Questionnaire short form (PFIQ-7) and the PFDI-20 will be used to measure the impact of prolapse on the patient’ s quality of life before surgery, as well as the degree of postoperative symptom improvement at 1 year and 3 years postoperatively [11]. For sexually active women, the Chinese version of the Pelvic Organ Prolapse/Urinary Incontinence Sexual Questionnaire short form (PISQ-12) will be administered [12]. The Patient Global Impression of Change (PGI-C) inventory will be administered to assess each subject’s perception of change of their prolapse condition after surgery using a 7-point Likert scale ranging from “much worse” to “much better”.

Perioperative parameters will be documented, including the operative time, estimated blood loss, length of hospital stay, postoperative pain score (visual analogue scales, VAS) and return to spontaneous voiding time. Perioperative complications will be recorded and scored according to the Clavien–Dindo classification.

The cost of admission is all of the hospitalization expenses, including the prescription drugs, laboratory and radiology, surgery and anesthesia fees, material fee and so forth.

Patients will visit the hospital at 3 months and annually after surgery. A physical examination including the POP-Q will be performed and complications will be recorded by a member of the research team blinded to the intervention. Mesh-related complications such as dyspareunia, pelvic pain and mesh erosion/complications will be categorized using the IUGA/ICS joint terminology CTS coding system. De novo dyspareunia is defined as those without baseline bothersome symptoms who developed bothersome dyspareunia during the follow-up time. De novo stress urinary incontinence is defined as those without baseline bothersome symptoms who developed bothersome stress urinary incontinence symptoms. For patients who do not show up for their postoperative appointments, telephone contact will be attempted. If they are contacted but refuse to continue to participate in the study, the reason for dropping out will be assessed.

### Interventions

At each center, all surgeries will be performed by physicians experienced with both surgical methods. In this study, all women with an intact uterus will undergo hysterectomy prior to mesh placement.

#### Modified self-cut mesh procedure

This surgical procedure will be performed according to the surgical technique that was described previously [5], which can be summarized as follows.

For the self-cut mesh procedure, a single piece of polypropylene mesh (TiLOOP®10 cm × 15 cm; pfm medical ag) will be cut into two parts for the anterior and apical compartment reconstructions. The anterior mesh includes four arms and a joint portion, and the apical mesh is composed of two rectangular strips. To reconstruct the anterior vaginal wall, a longitudinal incision will be made into the anterior vaginal mucosa starting at 3–4 cm cephalad to the urethral meatus and extending up to the vaginal apex. The vesicovaginal space will be dissected with both blunt and sharp separation until the bilateral obturator internus muscles and the arcus tendinous fascia pelvis (ATFP) are palpated at the level of the ischial spines. Using the obturator puncture needle designed and made for the self-cut procedure, the superficial arms of the anterior mesh will be advanced from an incision 1 cm proximal to the prepubic end of the ATFP to the skin incision at the level of the clitoris. The deep arm is then advanced from the ATFP 3–4 cm away from the ischial spine to a cutaneous incision 2 cm inferior and 1 cm lateral to the first incision. The four arms of the anterior mesh are drawn from the vagina to the perineum and the mesh is flattened into the vesicovaginal space below the bladder. The middle compartment and the posterior vaginal wall are then addressed. A mucosal incision is made in the midline posterior vagina from the level of the vaginal apex to approximately halfway down the posterior vagina. Sharp and blunt dissection continues laterally until the ischial spines and sacrospinous ligaments can be palpated on both sides. Skin incisions are made 3 cm lateral and 3 cm inferior to the anus on both sides. A needle is used to puncture through the anorectal fossa and then through the sacrospinous fascia and the spine fascia near the ischial spine. Rectangular strips of mesh are drawn from the inside to the outside, and the mesh strips are fixed to bilateral uterosacral ligaments. Tension-free placement is ensured before the mesh is trimmed at the skin. We close the vaginal mucosa and skin with absorbable sutures. We use traditional posterior colporrhaphy to repair the distal two-thirds of the posterior vaginal wall.

#### Mesh-kit procedure

This surgical procedure using the commercially available titanium-coated polypropylene mesh with six arms (TiLOOP®Total 6; pfm medical ag) is performed as follows. Insertion of the mesh is performed with tunnelers for the transobturator and ischiorectal passage. After colpotomy and preparation of the vesicovaginal fascia, the mesh is implanted according to the manufacturer’s advice. The anterior arms are inserted through the obturator fascia, the middle arms through the posterior angle of the obturator foramen and the posterior arms in the sacrospinous ligaments.

After tension-free implantation of the mesh, the colpotomy is closed using a continuous absorbable suture and vaginal packing is placed until the next morning. Prophylactic antibiotics are administered immediately before the procedure and for 3–4 days after operation according to the surgeon’s decision.

### Sample size and power considerations

The aim of the trial is to test the hypothesis that the procedure with self-cut mesh is non-inferior to the procedure with a mesh kit in terms of the composite success rate and safety. According to Fünfgeld et al.’s report, the anatomic success rate (POP-Q stage ≥ II, different from this proposed study) after 12 months across all compartments was 86% [8]. Based on a success rate of 90% in this study and 10% as the non-inferiority margin (β = 0.2 and one-sided α = 0.025), 284 patients (142 in each group) would be required. Taking into account 10% who do not continue to the 1-year follow-up visit, a total of 312 patients will be recruited. The procedure with self-cut mesh will be considered non-inferior if the lower limit of the 95% confidence interval in success rates lies above the non-inferiority margin of − 10%.

### Data analysis

We will analyze the data on an intention-to-treat basis. Frequency and percentages will be used to describe categorical variables, and means and standard deviations (SDs) or interquartile range used to describe normally distributed continuous data. We will use the chi-square test or Fisher's exact test to compare dichotomous outcomes between treatment groups. We will use the *t* test or Wilcoxon rank sum test to compare continuous outcomes between treatment groups. We will also use the paired *t* test for the before-and-after difference. If there are any important imbalance in the baseline characteristics between groups, we will use logistic regression adjusting for baseline covariates for binary outcomes, and use linear regression analysis adjusting for baseline variables.

For the primary outcome, we will also perform subgroup analysis based on the body mass index (BMI) (< 24 kg/m^2^ versus ≥ 24 kg/m^2^), history of POP procedure (primary versus recurrent) or prolapse stage (stage 3 versus stage 4). AEs will be listed and analyzed using a chi-squared test or Fisher’s exact test. Severe AEs will be listed and described in detail. Statistical significance is defined as two-sided *P* < 0.05.

### Ethics

This protocol and the consent forms have been reviewed and approved by the central institutional review board (IRB) of Peking Union Medical College Hospital prior to initiation of the trial (JS-1278). Ethical approval was not demanded at each center. No important protocol modifications have been made after approval.

### Data safety and monitoring

The Data Safety Monitoring Board (DSMB) in our study includes two clinicians experienced in pelvic reconstructive surgery and a statistician. The members of the DSMB will meet prior to the start of recruitment and at each interim meeting throughout the trial. During each meeting, they will evaluate adherence to the protocol and timeliness of recruitment. DSMB members will monitor the adverse effects, especially severe adverse events and mesh-related issues. On-site monitoring visits are planned to ensure the reliability and compliance to the protocol when needed.

A planned, masked, formal interim analysis will be performed after recruitment of half of the sample size. The DSMB has the right to stop the trial ahead of schedule through voting in the case of clearly demonstrated harm or benefit. Complications will be reported in the complication registration system in China. Patients who participated will be given post-trial follow-up every year. Patients with serious complications can be transferred to the principle investigator for the management of mesh complications if needed.

## Discussion

This study is a continuation of our previous research. We anticipate that the results of the trial will provide additional data regarding the safety and 1-year efficacy of transvaginal mesh repair. If we are able to show that the self-cut mesh procedure is non-inferior to the mesh-kit procedure, then it may be reasonable to recommend use of self-cut mesh due to the substantially lower cost for the patient.

### Trial status

The trial is ongoing. The protocol is version 1 and was completed on 4 January 2018. The trial was registered on 17 January 2018. The first patient was enrolled on 22 January 2018. The expected date of recruitment completion will be March 2020.

## Supplementary information

**Additional file 1.** SPIRIT Checklist: Recommended items to address in a clinical trial protocol and related documents.

## Data Availability

The datasets used and/or analyzed during the current study are available from the corresponding author on reasonable request.
